# Brief history of ophidiology and ophidism in Colombia

**DOI:** 10.1093/trstmh/trag051

**Published:** 2026-04-28

**Authors:** Carlos A Cañas, Santiago Castaño-Valencia, Fernando Castro-Herrera

**Affiliations:** Unit of Rheumatology, Fundación Valle del Lili, Cali 760031, Colombia; CIRAT: Centro de Investigación en Reumatología, Autoinmunidad y Medicina Traslacional, Universidad Icesi, Cali 760031, Colombia; Laboratory of Herpetology and Toxinology, Department of Physiological Sciences, Department of Health Sciences, Universidad del Valle, Cali 760031, Colombia; Laboratory of Herpetology and Toxinology, Department of Physiological Sciences, Department of Health Sciences, Universidad del Valle, Cali 760031, Colombia

**Keywords:** envenoming, evolution, herpetology, snake, snakebites, venoms

## Abstract

This review traces the historical trajectory of ophidiology (the study of snakes) and ophidism (snakebite envenoming) within the Colombian context. Despite the profound cultural and scientific significance of the human–serpent relationship in the region, the relevant literature remains fragmented, with foundational historical works neither digitized nor readily accessible. To address this, we conducted a narrative review of primary sources spanning from the pre-Colombian era to the present. Our methodology involved sourcing material from specialized libraries, historical archives, personal collections and contemporary scientific databases. The analysis identifies and contextualizes pivotal contributions across distinct historical periods, from early archeological evidence and colonial accounts to foundational eighteenth-century works and the formalized scientific studies of the last two centuries. The review culminates by highlighting how modern Colombian research, including significant international collaborations, is now producing influential work on snake biology, venoms and envenoming that commands global attention.

## Introduction

The interplay between humans and snakes in Colombia encompasses a complex duality, reflected in the parallel scientific disciplines of ophidiology (the study of snake biology, ecology and evolution) and ophidism (the study of snakebite envenoming). This relationship, rooted in deep evolutionary history and cultural perception, has generated a substantial yet dispersed corpus of knowledge. A comprehensive historical analysis of these fields within the Colombian context remains absent, hindered by the fragmented and often inaccessible nature of primary sources. Much of the foundational literature, although critically relevant, exists outside digital repositories, residing in personal collections and specialized libraries, thus posing a significant barrier to scholarly synthesis.

This review aims to consolidate and narrate the historical development of ophidiology and ophidism in Colombia from the pre-Columbian period to the present day. The objective is to construct a coherent timeline that identifies pivotal works, contextualizes them within their historical and scientific milieus, and traces the evolution of thought and practice. To achieve this, we employed a narrative review methodology based on an exhaustive search of heterogeneous sources. Historical data were retrieved from archaeological reports, colonial chronicles and pre-twentieth century scientific publications housed in national repositories, including the Luis Ángel Arango Library, the National University of Colombia and the National Institute of Health. For the modern period, systematic searches were conducted in the PubMed, Scopus and LILACS databases to incorporate contemporary scientific literature.

The narrative is structured chronologically. It begins with an examination of the geographical and biodiversity context of Colombia, providing a foundational understanding of the ophidian fauna. It then explores the evolutionary and early cultural relationships between humans and snakes, drawing on archaeological artifacts and first-contact accounts. Subsequent sections analyze the influence of European Enlightenment thought on eighteenth-century naturalist observations, followed by a critical review of the formalized scientific contributions from the nineteenth and twentieth centuries. The review concludes by highlighting current research trends, underscoring how modern Colombian science is contributing to global advancements in venom biology, antivenom therapy and snake ecology. Through this synthesis, we aim to provide a definitive historical framework that acknowledges past scholarship and illuminates the trajectory of future research.

## Biogeography of Colombia

Colombia is a tropical country in northern South America, bordered by two oceans: the Atlantic Ocean to the north and the Pacific Ocean to the west. It borders Venezuela to the northeast, sharing the desert region of ‘La Guajira’ to the north and the grasslands known as ‘Llanos Orientales’ to the east. It borders Brazil to the southeast and shares the Amazon; and Ecuador and Peru to the south, sharing the biogeographic Chocó region to the west and the Amazon to the east. It has three mountain ranges with different thermal zones corresponding to the final trifurcation of the Andes, in addition to a high mountain range to the northeast corresponding to the Sierra Nevada de Santa Marta. Various river basins are distributed throughout its territory, including those of the Amazon and Orinoco rivers, and those of the inter-Andean valleys of the Magdalena and Cauca rivers.^1^

The noted geographical features determine several habitats including tropical rainforest, tropical dry forest, montane rainforest (cloud forest), high montane rainforest (including páramos), grasslands, scrublands, savannas, deserts, swamps, wetlands, major rivers, estuaries, beaches, rocky coasts and mangroves,^2^ making it one of the most biodiverse countries in the world.^3^ With only 1% of the Earth’s surface, it has a little more than 10% of the planet’s biodiversity. Around 270 species of snakes have been recorded, corresponding to ∼8% of the world’s diversity, placing this country among the 10 with the greatest number of ophidians.^4^ Thirty are elapids and 19 vipers, distributed throughout almost the entire territory, except in the waters of the Caribbean and in the highlands >3500 m above sea level. Numerous snake species or genera are distributed across both Central America and northern South America.

This review traces the historical trajectory of ophidiological knowledge in Colombia, charting its evolution from early cultural representations to contemporary interdisciplinary science. The narrative begins in the pre-Columbian era, evidenced by millennia-old rock art and artifacts depicting serpents, which establish the deep-rooted symbolic and practical relationship between humans and snakes in the region.

A pivotal transition occurred during the proto-scientific period of colonial chronicles (sixteenth–eighteenth centuries), when European accounts provided the first written, although often myth-tinged, descriptions of local fauna and envenoming. This era gradually gave way to the foundational scientific period of the late eighteenth and early nineteenth centuries, characterized by Enlightenment-driven expeditions and the first systematic, Linnaean classifications of local snakes.

The formal institutionalization of the field in the nineteenth century, through museum collections and early clinical studies, established the basis for modern ophidiology and toxinology. This trajectory culminates in the twentieth and twenty-first centuries with the consolidation of specialized research programs, the advent of venomics and antivenomics, as well as the integration of ecology, clinical medicine and biochemistry, positioning Colombian ophidiology within a global scientific context.

## The evolutionary roots of the human–serpent dynamic

It is important to situate humans and snakes evolutionarily to better understand their biological interaction. The Earth was formed ∼4.56 billion years ago,^5^ and life ∼3.7–3.8 billion years ago.^6^ The origin of amphibians is estimated to have been ∼370 million years ago, from finned fish that were structures into pentadactyl limbs that were used to move on land; a fossil with these characteristics is *Ichthyostega*.^7^ The oldest reptile fossil that has been reported is *Hylonomus lyelli*, which dates back 312 million years.^8^

The origin of crown-group snakes, as estimated by molecular clock analyses, likely occurred during the Late Cretaceous period, ∼145–66 million years ago.^9^ Subsequent geological events, notably the break-up of the supercontinent Pangea,^10^ facilitated the divergence of early crown-group populations into western (American) and eastern (Eurasian) lineages.

The fossil record, however, reveals that more basal, stem-group snakes predate this crown-group radiation. The oldest currently known snake fossil is *Eophis underwoodi*, dated to ∼167 million years ago from the Middle Jurassic, discovered in African deposits.^11^ This indicates that the snake lineage had already diverged from other squamates well before the estimated emergence of the modern crown group.

One of the most notable fossil snakes from South America is *Titanoboa cerrejonensis*, recovered from the Cerrejón Formation in La Guajira, Colombia. Dating to the Paleocene epoch ∼60 million years ago, this colossal snake is estimated to have reached lengths of ∼13 m and a mass of ∼1135 kg.^10^ The gigantism exhibited by *Titanoboa* is frequently attributed to high paleotemperatures during this period, which would have been particularly favorable for large-bodied poikilothermic animals.

Primates (Order Primates Linnaeus, 1758) later split into the parvorder Platyrrhini in America (E. Geoffroy, 1812) and Catarrhini in Africa (E. Geoffroy, 1812); from the latter, *Homo sapiens* emerged ∼200 000 years ago.^9^

*Homo sapiens* was originally associated with snakes in Africa and later Eurasia during migrations. Their encounter with American snakes occurred when humans migrated through these territories during the last Ice Age, crossing the Bering Strait 28000 years ago and began to be distributed in the American continent 16000 years ago, based on dental, linguistic and genomic studies.^10^ Traces of human presence in Colombian territories date back ∼16 000 years.^11^ The reunion of humans from the old and new continents occurred in 1492 with the discovery of America, although earlier encounters are hypothesized.^12^ Europeans, upon interacting with Indigenous peoples, also encountered diverse flora and fauna, including snakes.^13^ They were particularly intrigued by the rattlesnake (*Crotalus* sp.), notable for its characteristic tail rattle.^14^

## Before the Spanish conquest

Before the conquest of the territories that correspond to Colombia, the aborigines marked their encounters with snakes in rock art figures (drawings made with pigments) and petroglyphs (forms of stone carving), some of which are still preserved. Representative examples of rock art are open-air figures in the La Lindosa mountain range in the Guaviare region that may date back to ∼12 000 years,^15^ and petroglyphs found on rock structures in the Orinoquia, which are estimated to have been made ∼9000 years ago.^16^ For some writers, these drawings are testimonies of rituals that their ancestors wanted to capture.^17^ Between these forms of annotated pictorial art and the appearance of the oldest ceramics that can currently be analyzed, dating back ∼3000 years, there is a great gap in knowledge. The well-preserved ceramics and goldsmith pieces with references to snakes and that impress with their beauty and laboriousness are from (i) the Quimbaya culture, from the department of Quindío^18^; and (ii) from the Chibcha linguistic family, to which two pre-Columbian societies belong, the Muisca and the Tairona, the first from the departments of Cundinamarca and Boyacá, and the second the northeastern region,^19^ and which date back to ∼2000 years ago.

## From the sixteenth century to the eighteenth century

The earliest written accounts describing snakes in the territory of present-day Colombia and its neighboring regions originate from European conquistadors and colonizers, later followed by descriptive writings by Catholic missionaries and travelers with various motivations. Most of these sources from the beginning of the sixteenth century to the beginning of the eighteenth century were historical-literary writings whose publications had to be authorized by European monarchical delegates assigned for that purpose. This is the case of the Spanish monarchy, which created the Royal Council of the Indies, an entity in charge of reading manuscripts, making a critical analysis of a religious and political nature, and in accordance with the European culture of the time, which led to cuts, censorship or modifications. Many of these sources constitute the so-called Chronicles of the Indies, which were subjected to these filters, and yet they constitute true literary gems and make a great contribution.^20^ Some European travelers came to America with economic motivations such as the extraction, transportation and commercialization of natural resources. Such is the case of the controversial Dutch East India Company, which sometimes brought delegates with the purpose of making descriptions of flora and fauna.^21^

The foundational work of systematically documenting and describing New World snakes belongs to Gonzalo Fernández de Oviedo (1478–1557). A native of Madrid who served as a soldier, navigator and official chronicler of the Indies, Oviedo produced the first comprehensive natural history accounts of these reptiles. His observations were informed by first-hand experience, beginning with his arrival in the Americas in 1513 as part of the pivotal expedition led by Pedrarias Dávila (1468–1531). This journey culminated in the establishment of Santa María del Darién, a settlement strategically positioned in the region that now comprises the border between Colombia and Panama, where Oviedo began his meticulous chronicles of the novel fauna. He was also a captain in the armies of Emperor Charles V.^22^ His best-known work was *Sumario de la Natural Historia de las Indias* (‘Summary of the Natural History of the Indies’), published in 1526 as part of his great work *Historia General Natural de las Indias, Islas y Tierra Firme* (General Natural History of the Indies, Islands and Tierra Firme), a compendium of his travels to this continent, where he remained for 22 years.^23^ Oviedo’s chronicles are widely regarded as the earliest and most valuable accounts from European chroniclers in the Americas. His detailed ethnographic contributions include descriptions of Indigenous dwelling construction techniques, such as elevated stilt houses and the use of hammocks, adaptations explicitly noted for their utility in avoiding encounters with dangerous fauna, including snakes.^24^

**Figure 1:**
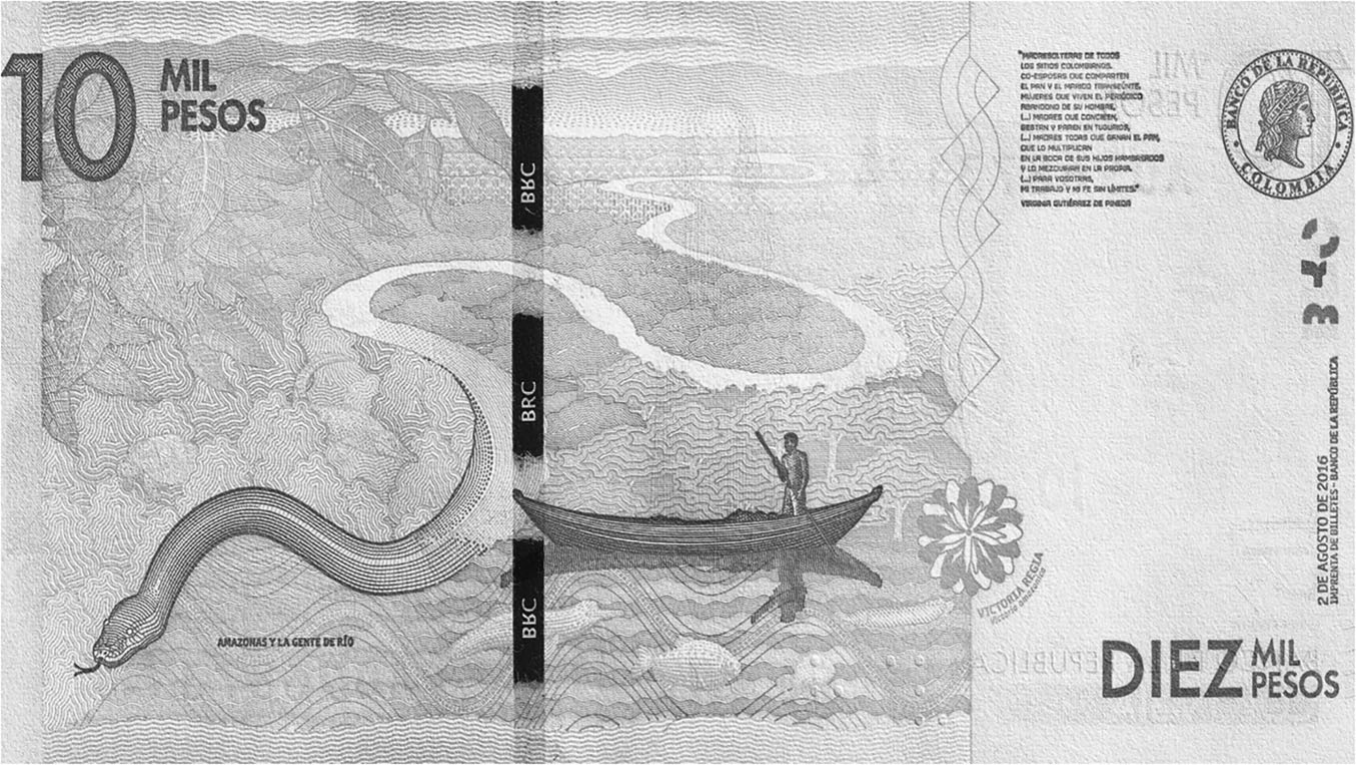
A 10 000 Colombian peso banknote evoking the myth of the great serpent that gives rise to the Amazon River and, with it, the origin of life. This myth originated among the ancient peoples who lived on its banks and is still maintained today.

In 1541, the Spanish explorer Francisco de Orellana (1511–46) led the first documented European expedition to traverse the entire length of the Amazon River, navigating from its Andean headwaters to its Atlantic estuary. The river’s principal channel courses through present-day Peru, Colombia and Brazil, while its vast drainage basin—the largest in the world—encompasses nine South American nations: Brazil, Peru, Bolivia, Colombia, Ecuador, Venezuela, Guyana, Suriname and French Guiana. He discovered its meandering course, which had already caught the attention of the natives and had linked its origin to an anaconda snake (*Eunectes murinus*), to which they had also attributed the origin of life, as well as a permanent connection between the underworld and the visible world. The writers William Ospina^25^ and Wade Davis^26^ have masterfully described these myths and legends. The Colombian 10 000-peso banknote features a representative image of the river’s association with *E. murinus* (Fig. 1).

**Figure 2:**
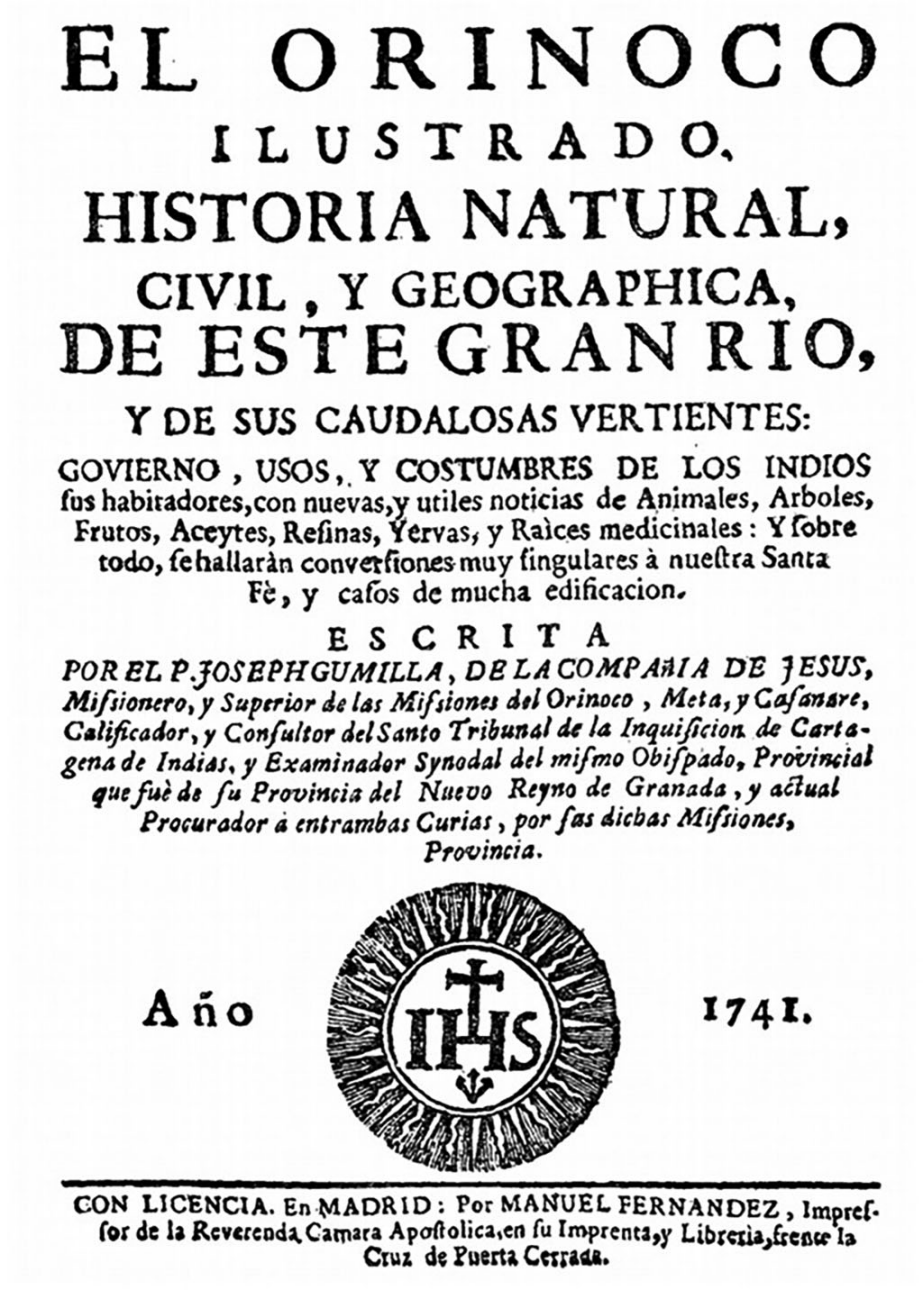
El Orinoco Ilustrado. A work by the Jesuit missionary Joseph Gumilla, a result of his evangelizing mission in ancient Venezuela and the New Kingdom of Granada. It provides comprehensive descriptions of the snakes of the Orinoco and treatments for their bites. The book was highly appreciated and widely read at the time, as evidenced by three editions: the first in 1741, and the other two in 1745 and 1791.

Later, some Spanish missionaries, mainly Jesuits, Franciscans and Augustinians, who were sent on an evangelizing mission, recounted their personal experiences, their interactions with the natives, their studies of their languages, and descriptions of the geography, flora and fauna, including snakes. Their relationship with these new lands was not easy, as they had to endure extreme climatic conditions and difficulties communicating with the natives, often in an atmosphere of violence and mutual suspicion. The missionaries' literary works provide descriptions of some snake species, which are frequently linked to notes with magical characteristics. The eighteenth-century exploration of the Orinoco River basin was significantly advanced by the work of the Jesuit missionary Joseph Gumilla (1686–1750). His seminal text, *El Orinoco Ilustrado* (1741), provides a detailed account of this major South American river system, whose transnational basin originates in Venezuela and, along a portion of its course, forms the natural border with Colombia^27^ (Fig. 2); in the publication he details the characteristics of a considerable number of snakes, such as hunters (species of the Colubridae family), boas (*Boa* sp.), rattlesnakes (*Crotalus* sp.), vipers (*Bothrops* sp.) and corals (*Micrurus* sp.), as well as the ‘contras’ or natural antidotes used by the natives to control their envenoming. He also describes the processing of plants that served to repel snakes.

Other valuable descriptions are attributed to Fray Juan Serra (also known as Fray Juan de Santa Gertrudis) (Mallorca, 1724–99). In 1757 he traveled to South America as a Franciscan missionary and during 1758–67 he founded a village in the Putumayo region called Agustinillo. The Franciscan friar Juan de Santa Gertrudis provides a valuable observational account from his eighteenth-century journeys through the southern provinces of the New Granada Viceroyalty, including Quito (present-day Ecuador), Popayán and Santafé de Bogotá. Following his return to Spain, he authored *Las maravillas de la naturaleza* (‘The Wonders of Nature’), a work notable for its ethnographic detail and for presenting a perspective on colonial life distinctly different from that found in official administrative records or earlier conquest chronicles.^28^

The Dutch naturalist Arnout Vosmaer (1720–99) conducted zoological observations in the Americas under the auspices of the Dutch West India Company. His 1767 publication, *Histoire Naturelle*, contains notably detailed and artistically refined accounts of New World fauna, including a particularly meticulous scientific description and illustration of the South American rattlesnake, *Crotalus durissus*.^29^

In 1889, the Dominican missionary Fray José de Calasanz Vela (1840–1919) traveled through the eastern region of Colombia with the objective of catechizing indigenous communities. During his expedition, he documented the herpetofauna encountered, notably recording observations on the local abundance of snakes. In his accounts, he particularly emphasized the prevalence of the boa (*Boa constrictor*), noting an illustrative instance where several specimens were observed occupying a single tree.^30^

The eighteenth century in Europe was marked by the Enlightenment, a cultural and intellectual movement that ushered in profound transformations in society at the time. This was the era of the French Revolution and philosophical movements such as Rationalism.^1^ From a scientific perspective, many innovative aspects were presented, including a scientific movement aimed at the classification of living beings, the starting point for important developments such as the theory of evolution. Of note are the contributions of Carl Linnaeus (Sweden, 1707–78) and Georges-Louis de Buffon (France, 1707–78), whose stories are masterfully covered in Jason Roberts' book, *Every Living Thing: the Great and Deadly Race to know all Life*.^31^ These Enlightenment currents significantly influenced scientific thought and natural history practices in the Americas.

This period, perhaps best described as protoscientific, represents a complex transitional phase in which emerging empirical methods coexisted and intertwined with literary narrative forms and ancestral knowledge, as well as religious and political frameworks.

## End of the eighteenth century and beginning of the nineteenth century

The years preceding the independence of the American nations including Colombia (late eighteenth and early nineteenth centuries) were very scientifically dynamic; notable from this period were the contributions of the Botanical Expedition of the New Kingdom of Granada led by the Spanish botanist and astronomer José Celestino Mutis (1732–1808),^32^ the writings of Alexander von Humboldt in his masterpiece initially published in French, *Voyage aux régions équinoxiales du nouveau continent* (‘Travels to the Equinoctial Regions of the New Continent’) (1814),^33^ as well as the contributions of the independence martyr Jorge Tadeo Lozano^34^ and the Franciscan religious Fray Diego García.

**Figure 3:**
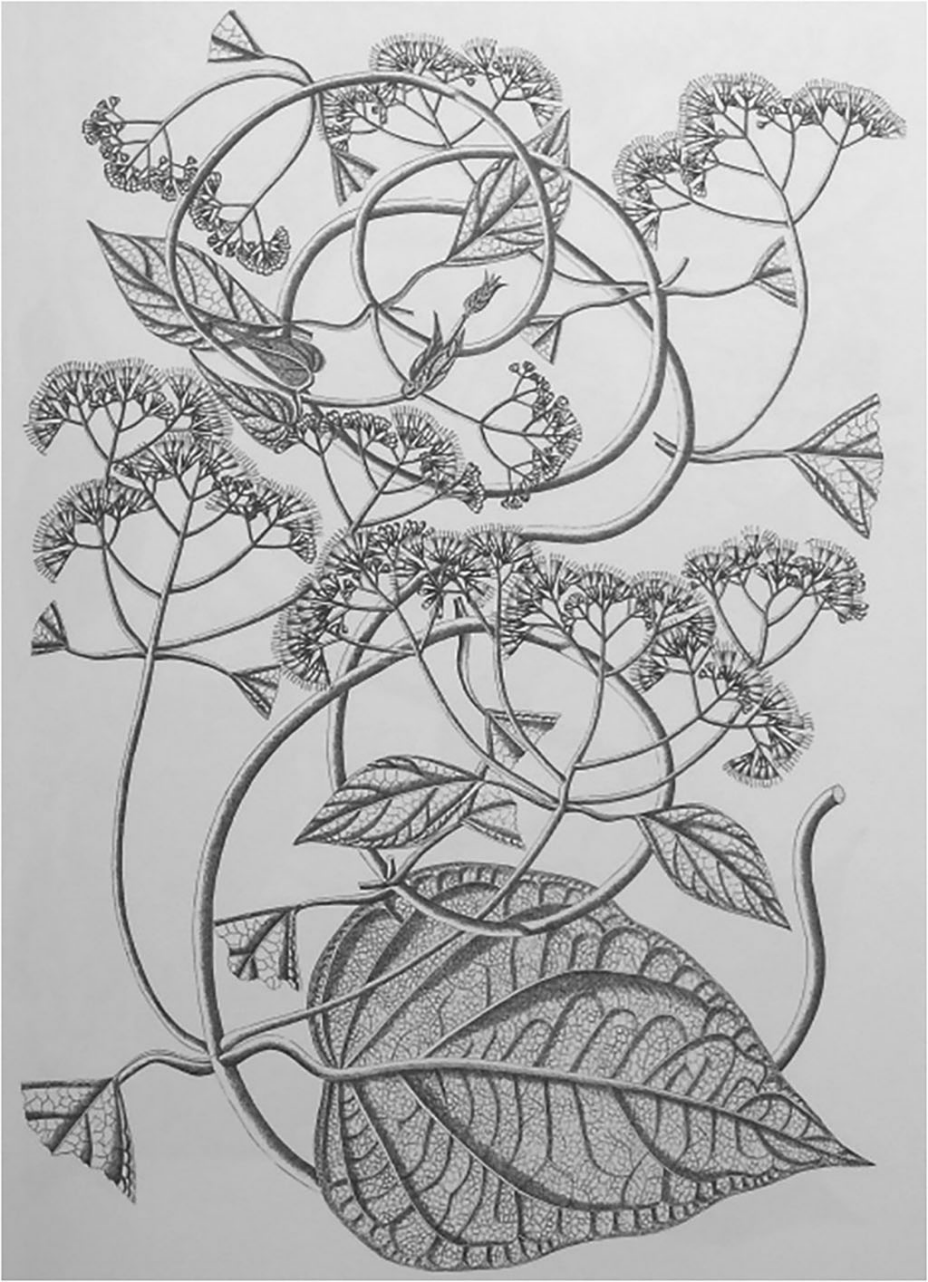
FJ Matís. ‘Guaco contra benenos (sic) de culebras’ (Guaco against the venomous snakes of Cauca).

In his *Diario de Observaciones* (‘Journal of Observations’), maintained from 1760 to 1790, José Celestino Mutis documented encounters with venomous snakes. Among his ethnobotanical records, he specifically described the use of the plant ‘Guaco’ (identified as *Mikania* sp.) from the region of Mariquita (Tolima), noting its local application as an antidote for snake venom.^35^ This plant was also studied by others such as Francisco Javier Matiz (1763–1851), who made very detailed drawings of it^36^ (Fig. 3), and Pedro Fermín de Vargas y Sarmiento (1762–1813), a naturalist and disciple of Mutis.^37^

In his seminal work, *Voyage aux régions équinoxiales du nouveau continent*, Alexander von Humboldt (1769–1859) provided detailed descriptions of numerous snake species based on observations made during his expedition to the Americas from 1799 to 1804. These systematic accounts significantly contributed to the development of natural sciences, providing foundational data for the emerging field of herpetology.

A notable contributor to the Botanical Expedition of the New Kingdom of Granada was the polymath Jorge Tadeo Lozano (Santafé de Bogotá, 1771–1816). His scholarly output includes the work *Memoria sobre serpientes* (‘Treatise on Snakes’), which was published in the journal *Semanario del Nuevo Reino de Granada* by Francisco José de Caldas in 1808.^38^ This work synthesizes contemporary knowledge regarding the taxonomic classification of snake species using the Linnaean methodology. Among the described species are the ‘*buio*’ (*Boa constrictor*), several snakes of the genus *Coluber*, the rattlesnake (*Crotalus durissus*), coral snakes (*Micrurus* sp.) and ‘*equis*’ vipers (*Bothrops* sp.). Lozano’s scientific rigor is evidenced in his formulation of a systematic research program to resolve outstanding questions regarding venomous snakes. His proposed methodology included the comparative anatomical study of species to determine venomous characteristics; experimental investigation into the activity and physiological effects of their venoms; chemical analysis of the venom’s composition; and empirical evaluation of traditional antidotes, such as the widely used ‘Guano’.

A key contributor to the Botanical Expedition was Fray Diego García (1745–94), a Franciscan monk from Cartagena. During his 7-year collaboration, he produced systematic records focusing on the zoology, botany and mineralogy of the Upper Magdalena Valley, work for which scholars regard him as Colombia’s pioneering zoologist and botanist.^39^

## Mid- and late-nineteenth century

Nineteenth-century naturalists and physicians, including Colombian and foreign researchers, documented snake biology and envenomation in scholarly publications. Naturalists undertook numerous expeditions, collecting biological specimens now preserved in global institutions. Colombia’s National Museum was established in 1822. Its creation stemmed from an initiative by Francisco Antonio Zea (1766–1822), a Colombian scientist and diplomat who, while serving as an ambassador of Gran Colombia to the UK, commissioned a French scientific delegation for the project.^40^

The French naturalist and collector Justin Goudot (1802–48), based in Bogotá, served as a correspondent for the Muséum National d’Histoire Naturelle in Paris from 1822 to 1842. His fieldwork was focused on assembling a herpetological collection, primarily from the Magdalena River Valley. Goudot participated in several major expeditions, including those directed by Jean-Baptiste Boussingault (1801–87) and François D. Roulin (1796–1874) to sites such as Cúcuta, Pamplona, Santa Rosa, Tunja and Bogotá. A significant collaboration occurred in 1824 with the Peruvian naturalist Mariano Eduardo de Rivero y Ustáriz (1798–1857), with whom he worked in the Bogotá and Vélez regions. The following year, their joint research extended to Mariquita, from which Goudot proceeded through Herveo towards Supía, and into the provinces of Antioquia, Popayán and Tolima.^41^

Throughout the mid-nineteenth century, the taxonomic cataloging of neotropical herpetofauna was advanced by several key European and American naturalists. From 1836 to 1854, the French zoologists André Marie Constant Duméril (1774–1860), his son Auguste Duméril (1812–70), and their collaborator Gabriel Bibron (1806–48), conducted systematic work that described numerous new reptile and amphibian species.^42^ Subsequent contributions came from the American herpetologist and ichthyologist Edward Drinker Cope (1840–97), who, from 1862 to 1899, studied specimens collected from Colombian localities, including the Turbo region (a zone shared with Panama) and Cartagena. Earlier, in 1858, the German naturalist Oskar Schmidt (1823–86), a botanist, zoologist and explorer, had examined relevant specimens held in the collection of the National Museum in Krakow.^43^

In 1870, Silvestre B. Higgins, an American engineer, homeopath and ophiologist based in Norosí (Bolívar), published *Culebras y reptiles venenosos* (‘Venomous Snakes and Reptiles’), later cited by Evaristo García in his manuscript *Los ofidios venenosos del Cauca* (‘The Venomous Snakes of Cauca’). The book begins with a table of the reptiles found in Colombia, mentioning some of their behaviors and ecological adaptations; it then refers extensively to the most well-known reptiles in the country and the clinical symptoms produced by the bite of different snakes.^44^ During 1875–6, the French botanist and explorer Édouard-François André (1840–1911) undertook an expedition through the Andes, where he systematically recorded the region’s flora and fauna. André, who was also a prominent architect and landscaper famed for designing parks in Monte Carlo, Montevideo and Luxembourg, contributed significant field observations during this journey. In 1861, the French physician and botanist Charles Saffray (1833–90) undertook a scientific expedition to Colombia, where he systematically documented the country’s native flora and fauna, with particular attention being paid to its ophidian species. There is a collection of literature and exceptional engravings from these two travelers in the book *Geografía pintoresca de Colombia*—*La Nueva Granada vista por dos viajeros franceses del siglo XIX* (‘Picturesque geography of Colombia—New Granada as seen by two French travelers of the nineteenth century’).^45^

George Albert Boulenger (1858–1937), a Belgian–English zoologist and polyglot, served as a leading taxonomist at the British Museum (Natural History). His extensive work included describing and providing scientific nomenclature for a vast number of species worldwide, with significant focus on snakes and fish. He studied various Colombian snakes based on collections by W. F. Resenberg, M. G. Palmer and H. G. F. Spurell.^46^ He compiled a detailed catalog of Colombian snakes housed in the British Museum.^47^

At the end of the nineteenth century, several contributions to Colombian medicine were made on snake envenoming by physicians in Medellín (Antioquia). The first description of a patient bitten by a venomous Colombian snake was made in 1887.^48^ In 1889, Andrés Posada Arango (1839–1922), a physician from Antioquia, published a review of the venomous snakes of northwestern Colombia.^49^ The physician Manuel Uribe Ángel (1822–1904), called at that time the ‘Father of Antioquia Medicine’, wrote in 1892 about the methods of neutralizing snake venom by means of gold chloride, a compound employed shortly before by Albert Calmette in France against European vipers.^50^ It is worth noting that, in the last decade of the nineteenth century, the study of antivenoms for snake envenoming began, based on the immunization of equines and the application of their plasma; initially in laboratory animals,^51^ then later in humans.^52^

**Figure 4:**
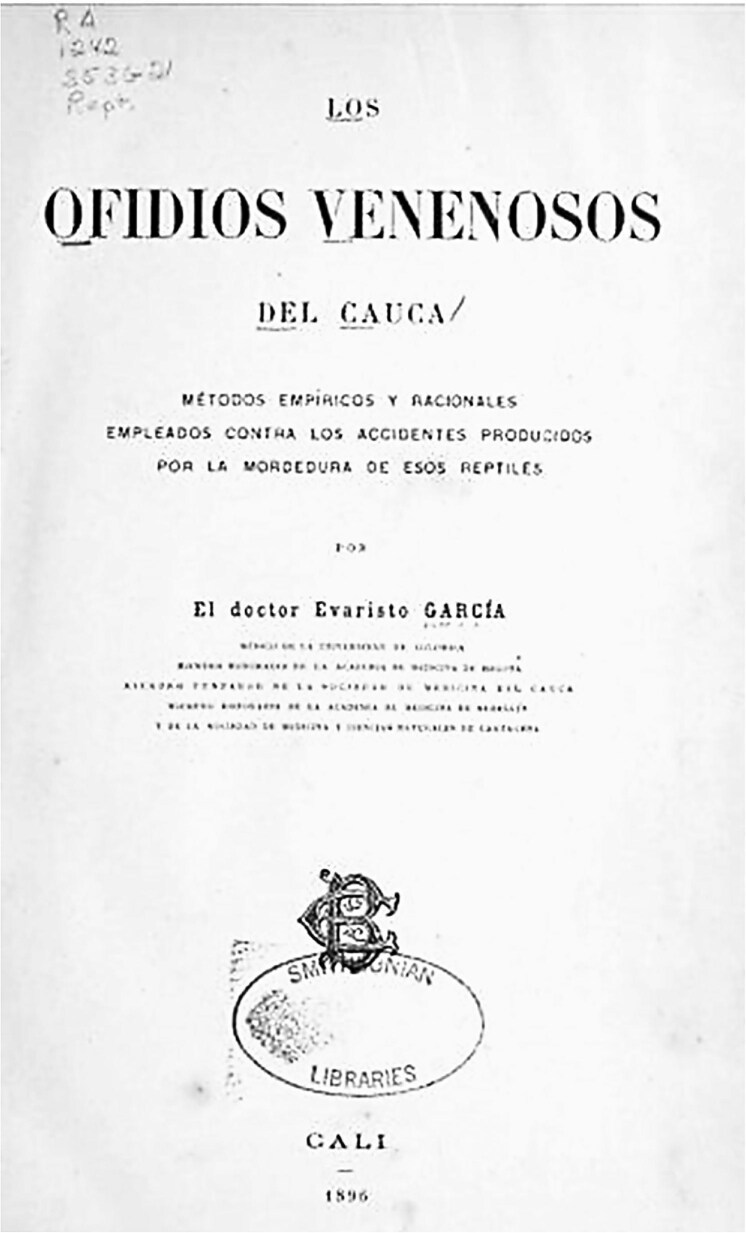
Cover of the book ‘Los ofidios venenosos del Cauca,’ by Evaristo García, published in Paris in 1896. (With permission for its publication given by the ‘Evaristo Garcia Foundation’ publishers).

**Figure 5:**
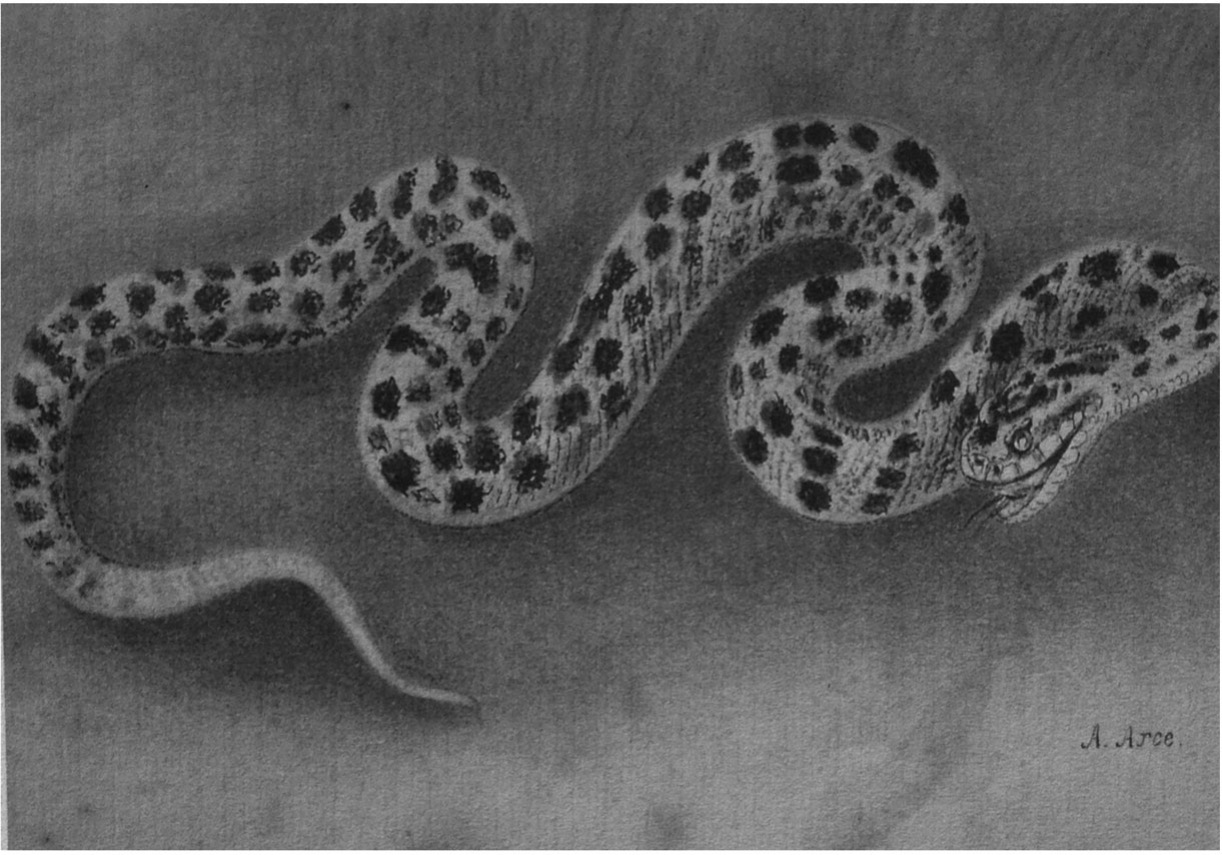
Illustration of the ‘*Lachesis punctatus*’ snake, the ‘rabo de chuca’ viper, from the book ‘Los ofidios venenosos del Cauca’ 1896. (With permission for its publication given by the ‘Evaristo Garcia Foundation’ publishers).

Evaristo García (1845–1921), a physician from Valle del Cauca who graduated from the National University of Colombia, was co-founder in 1873 of the Society of Medicine and Natural Sciences of Bogotá, and founder in 1887 of the Society of Medicine of Cauca,^53^ where in the solemn session of 20 July 1892, the doctors Luis J. Uricoechea and P. P. Scarpetta presented the monograph *Los ofidios venenosos del Cauca* (Fig. 4), which was later published in Paris in 1896.^54^ This book was translated into French and read by Professor A. Laveran, a member of the Academy of Medicine of Paris, and winner of the Nobel Prize in Medicine in 1907 for the discovery of the causative agents of malaria and trypanosomiasis, who commented: ‘It is the most complete and extensive work that in French or in a foreign language, has appeared until today on venomous animals and envenoming’. E. García described a new species of snake, the ‘Rabo de Chucha’ of Chocó, which he named *Lachesis puntactus*. This description remained forgotten until it was corroborated by Brother Nicéforo María (1929) and Emmett Reit Dunn (1944). It is currently called *Bothrops punctatus* (García–1886)^55^ (Fig. 5).

Other notable contributors at the end of the nineteenth century are mentioned below. Heinrich Otto Wilhelm Bürger (1865–1945), a German zoologist, conducted expeditions through several regions of Colombia in 1900, documenting for the regions of Honda, La Dorada, Bogotá and Villavicencio.^56^ W. F. Rosenberg, M. G. Palmer and T. H. Barbour amassed a significant collection of new amphibians and reptiles from the regions of Buenaventura, Cali and Noamaná, and from the basins of the San Juan, Peña Lisa and Condoto rivers.^57^ Consul Friedrich Carl Lehmann, a native of Popayán, dedicated himself to studies of the fauna and flora of Alto Cauca and Nariño, and made expeditions along the Pacific Coast between the Mira and Patía rivers, where he drowned. He collected many new species of amphibians and reptiles, which were published by Oskar Boettger.^58^ O. Boettger also published herpetological material collected by Geor Hübner in 1895 at the Inírida River, and by Fritz Regel in 1897 in the Magdalena Valley.^59,60^ Several collections of snakes gathered by various explorers were published by F. Werner,^59^ O. Fuhrman and M. G. Peracca.^61^

The early scientific study of ophidiology was fundamentally rooted in anatomy and taxonomy. This foundational work preceded and enabled a pivotal transition towards modern biological paradigms, where understanding became structured around the physiological functions of cells and organs, as well as biochemical principles. Concurrently, therapeutic approaches advanced significantly with the parallel development of antibiotics and the critical innovation of specific antivenoms.

## Early twentieth century

Many scientists made contributions to different fields of Colombian ophidiology in the first half of the twentieth century, including E. R. Dunn, the Christian brothers Apolinar María and Nicéforo María, Karl Patterson Schmidt and Afradio Do Amaral.

E. R. Dunn (1894–1956) was an American herpetologist who studied species from the USA, Panama and several South American countries. He was in Colombia from June 1943 to July 1944 through the Nelson Rockefeller Committee’s Inter-American Cultural Exchange Program, compiling and publishing his studies in the journal *Caldasia* in 1944.^62^ In another study, he refers to snakes from the Bogotá area.^63^ He and his wife worked extensively with specimens collected by E. D. Cope. Several names given to species of snakes refer to his name, such as *Atractus dunni* (Savage 1955) (Dunn’s ground snake), *Geophis dunni* (K. P. Schmidt 1932) (Dunn’s earth snake), *Hydromorphus dunni* (Slevin 1942) (Dunn’s water snake) and *Porthidium dunni* (Hartweg and J. A. Oliver 1938) (Dunn’s hognose pitviper).

Two naturalists belonging to the Hermanos Cristianos Community, Apolinar María (Bogotá, 1909–Bogotá, 1988) and Nicéforo María (Lavoûte-Chilhac, France, 1888–Fusagasugá, Colombia, 1980), compiled a census of Colombian snakes around the 1940s, recording 180 species and subspecies, 26 of which were found in the country for the first time.^64^ They established numerous contacts with universities and museums in several countries, including the Smithsonian Institution in Washington. They sent numerous animal samples for proper classification.

K. P. Schmidt (1890–1957), an American herpetologist, trained at Cornell University. From 1916 to 1922, he worked as a scientific assistant in herpetology at the American Museum of Natural History in New York. From 1923 to 1934 he made several expeditions to collect reptiles from Central and South America. From 1941 to 1955 he was curator of the Field Museum in Chicago. He provided descriptions of coral snakes (genus *Micrurus*) from northern South America.^65^ He studied collections of Colombian snakes from the Smithsonian Institution.^66^ He died of coagulopathy associated with envenomation from the bite of a ‘boomslang’ snake (*Dispholidus typus*), which had been sent for identification by the then director of the Lincoln Park Zoo in Chicago.^67^

Afradio Do Amaral (Belém, Brazil 1894–São Paulo, Brazil 1982) was a Brazilian physician and later herpetologist who practiced medicine briefly before leaving for São Paulo in 1917. He worked at the Butantan Institute, an organization in charge of producing antivenom, under the direction of Vital Brazil. He was director of this institute for several years. He moved to the USA, where he was Director of the Antivenin Institute of America and worked as a professor at the Harvard School of Public Health, where he received a doctorate in 1924.^68^ He classified new snakes in Colombia, based on material sent by Apolinar and Nicéforo María from 1923 to 1954. He had >450 publications and described 15 new genres and ∼40 species of snakes.^69^ Several species bear his name, including *Gymnodactylus amarali* (Barbour 1925), *Caaeteboia amarali* (Wettstein 1930) and *Mastigodryas amarali* (Stuart 1938).

In the first half of the twentieth century, it is also worth highlighting the publications of Brother Daniel María (1909–88), who was Director of the Colegio San José in Medellín,^70,71^ as well as the work of Hampton Wildman Parker, on the reptiles and amphibians of Gorgona Island.^72^ In 1917, Bernardo Samper Sordo and Jorge Martínez Santamaría, after studying in the USA and London aspects related to the preparation of biological agents such as vaccines and serums, founded the Samper-Martínez Laboratory in Bogotá. In 1926, the nation purchased the laboratory and in 1975 it was established as the Instituto Nacional de Salud, which, among many diagnostic functions and production of biologicals, develops the production of antivenoms.^73^ During the 1930s and 1940s, the production of antivenom was established, which required the development of the Serpentarium in the city of Armero, Tolima, which was partially destroyed in the avalanche caused by the eruption of the Ruiz volcano in 1985.

In the second half of the twentieth century, the contributions of Federico Medem (Riga, Latvia, 1912–Bogotá 1984) are noteworthy. He arrived in Colombia in 1950, a few years after leaving his country because of the Russian Revolution. He studied zoology at the Humboldt University of Berlin and then at the University of Tübingen. He was an instructor at the Institute of Zoology at the University of Bern, Switzerland. He earned his doctorate at Naples Zoological Station, where he worked under Gustav Kramer. He performed his military service on the Russian front and worked in postwar Germany and Switzerland. After arriving in Colombia, he worked with the Universidad de Los Andes and the Universidad Nacional de Colombia at a research station in Villavicencio. He was an active campaigner for species protection and produced several scientific publications, mainly on the taxonomy of reptiles, including snakes. He participated in joint explorations with Dr Santiago Rengifo Salcedo, Director of the Roberto Franco Institute in Villavicencio and a professor at the National University of Colombia.^74^ Several species of reptiles bear his name in his honor, including a coral species, *Micrurus medemi* (Roze 1967).

During the 1960s and 1970s, notable works were published, by Latvian-born Janis Roze (1926–), a professor at the City University of New York, who described various species of snakes from Venezuela in common with those from Colombia^75–77^; by Armando Dugand, who published the report ‘Serpentifauna of the Caribbean Plain’ in 1975^78^; and by the professors J. R. Tamsitt and D. Valdivieso, who, among other works, described the island snakes of the Colombian Caribbean.^79^

On 10 April 1948, a large part of La Salle Museum’s snake collection disappeared. In 1978, the National Institute of Natural Resources of Colombia (INDERENA) began regulating scientific collections (Decree 1608 of 1978). With the increase in zoological research, particularly herpetological research, amphibians and reptiles began to be deposited at La Salle Museum and the Institute of Natural Sciences (ICN). In 1950, Professor Hernando Osorno Mesa took charge of the ICN collections and managed to maintain and expand them. Later, with the creation of the Faculty of Biology at the Universidad Nacional de Colombia in 1965, several professors, including José Pablo Leyva, F. Medem, Humberto Alarcón and Pedro María Ruiz, consolidated the accumulation of snake specimens. More recently, Professor John D. Linch moved to Bogotá from Nebraska (USA).^80–82^

The Probiol Laboratory, founded by Cesar Gómez Villegas, a physician from the University of Antioquia with a specialization in biologics production at Harvard University, began operations in 1975. Since its inception, this pharmaceutical laboratory has been very important for producing antivenoms.^83^

The work of María Cristina Ardila Robayo (1947–2017), a Colombian herpetologist and professor at the Universidad Nacional de Colombia based in Bogotá, who worked in the university’s Natural History Museum,^84^ deserves a special mention. In 2003, she compiled a collection of specimens from the ICN Laboratory’s collections.^85^ Currently, there are recognized herpetological collections at the Universidad de Antioquia, Universidad del Valle and Universidad del Cauca, in the museums of the Universidad de La Salle and the Colegio de San José in Medellín, in addition to some emerging ones at the Universidad Industrial de Santander, the Universidad Javeriana of Bogotá and the Universidad del Tolima. The collection of the former INDERENA was transferred to the Alexander von Humboldt Institute.^86^

## Late twentieth century and twenty-first century

Scientific inquiry into venomous snakes and ophidism in Colombia during recent decades has progressed across several interconnected fronts. Research has advanced significantly through the detailed clinical characterization of envenomation syndromes, the application of venomics and antivenomics to understand venom composition and antivenom efficacy, as well as via the systematic evaluation of ethnobotanical antidotes. Concurrently, investigations exploring the biomedical potential of venom components have emerged alongside continued contributions to taxonomic clarification.

The clinical characterization of envenomation by genera has been described nationwide,^87^ with regional analyses, including by Daniel Pineda *et al*. on cases from the Eastern Plains and Amazon Basin^88^; Juan Silva-Haad on *Bothrops* spp. in the Amazon^89^; R. Otero *et al*. on envenomation in Antioquia and Chocó (tropical rainforest and Pacific lowland snakes)^90,91^; M. Sevilla-Sánchez *et al*. in Cauca^92^ and Nariño (Andean highlands)^93^; R. Badillo *et al*. in Santander (northeastern dry forests)^94^; and C. A. Cañas *et al*. in Valle del Cauca.^95^ Descriptions of patients with snakebite are also reported from the University of Tolima in Ibagué city.^96,97^

Key research groups include the Universidad de Antioquia’s Snake/Scorpion Envenomation Program,^98^ led by Rafael Otero, Sebastián Estrada Gómez and Jaime Andrés Pereañez Jiménez, and which is recognized for toxinology and captive venomous snake husbandry. The Universidad del Cauca’s herpetology group, under Santiago Ayerbe and Armando Javier Folleco-Fernández,^99^ focuses on ecological and epidemiological studies, including the description of *Bothrops ayerbei*,^100^ the cardiotoxic effects of *Lachesis acrochorda*,^101^ toxinological profile and histopathological alterations induced by *Bothrocophias campbelli* venom,^102^ among others. Contributions from Fernando Castro Herrera, Santiago Castaño and Carlos Alberto Cañas (Universidad del Valle and Icesi University) span clinical and ecological research.^55,103–111^ In Bogotá, researchers such as Cornelis Johannes Marinkelle^112^ and Enrique Rodríguez^113^ have advanced taxonomic studies.

Venomic studies led by Universidad de Antioquia, in collaboration with Juan José Calvete (Institute of Biomedicine of Valencia, Spain) and José María Gutiérrez (Clodomiro Picado Institute, Costa Rica), have mapped the proteomic profiles of Colombian snake venoms.^114–128^ The Universidad de Antioquia has presented its own studies, such as those related to *Micrurus* venoms.^129,130^ Universidad del Valle researchers characterized the biochemical properties of viperid toxins,^131–135^ complemented by works from Juan Manuel Renjifo,^136–138^ Salazar-Valenzuela *et al*.^139^ and Quintana-Castillo *et al*.^140^

Antivenom efficacy trials^141–150^ have evaluated both preclinical and clinical outcomes. Studies are beginning to be carried out on the enzymatic inhibition of venoms, based on in silico research.^151^

Snake venom-derived compounds show potential for diagnostics (e.g. coagulation assays),^152^ therapeutics^153^ (e.g. hypertension, autoimmune disorders),^154^ antineoplastic agents,^155–160^ antimicrobial peptides^161–164^ and antimalarial candidates.^165,166^

**Figure 6:**
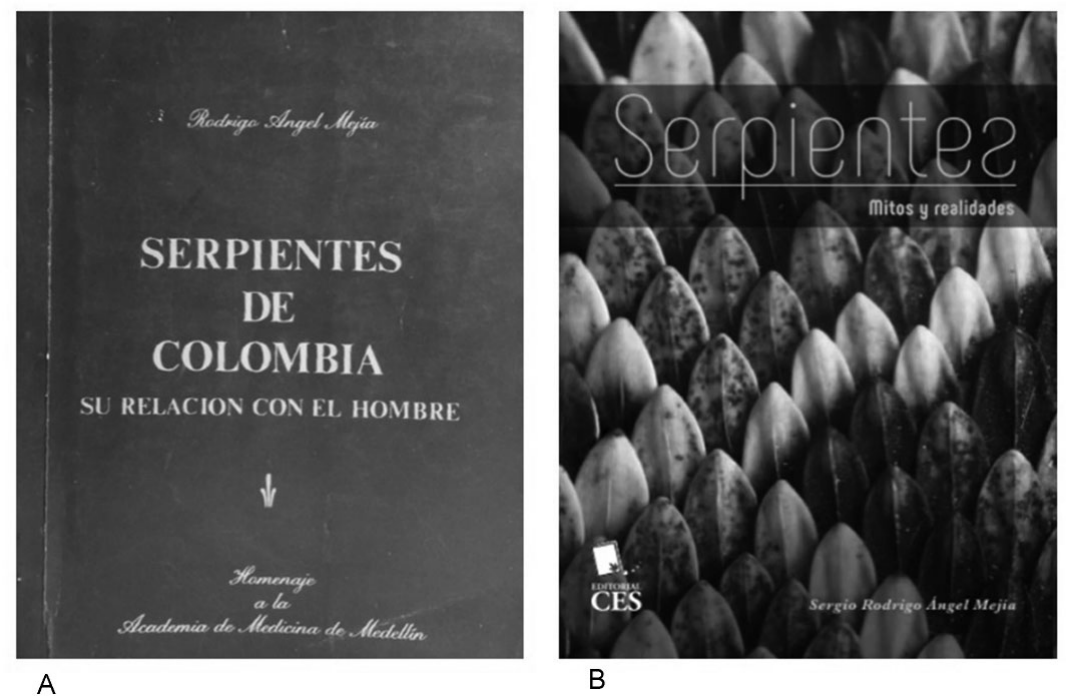
A: Cover of the book ‘Serpientes de Colombia. Su relación con el hombre’ by Rodrigo Ángel Mejía, published in Medellín by the Secretariat of Education and Culture of Antioquia in 1987, a book that has been a guide and reference in Colombia since its publication. **B:** Cover of the book ‘Serpientes. Mitos y realidades’ Rodrigo Ángel Mejía’s second book, published in 2017 by the CES University of Medellín Publishing House.

Notable publications include Rodrigo Ángel Mejía’s *Serpientes de Colombia: Human Interactions* (1987)^167^ (Fig. 6A) and *Snakes: Myths and Realities* (2017)^168^ (Fig. 6B); Alejandro Vázquez De Karzow’s *Venomous Snakebites* (1995)^169^; Rafael Otero’s primary clinical reference *Snakebite Diagnosis and Treatment Manual* (1994)^170^; and *Venomous Snakes: Lessons from Colombia* (2016)^171^ by Fundación Valle del Lili/Universidad del Valle. Jhonattan C. Campbell and William W. Lamar’s definitive text *Venomous Reptiles of the Western Hemisphere* (2004)^172–174^ and regional works by S. Ayerbe^175^ from Popayán, Hector Charry from Manizales,^176,177^ Luis F. Villota^178^ and Jorge Quiñonez from Cali,^179^ Ronald A. Diaz-Flórez from Bogotá^180^ and regional corporations,^181,182^ further enrich Colombian ophidiology. In 2024, Juan M. Renjifo donated >30 000 photographs of Colombian wildlife, including snakes, collected in the second half of the twentieth century and two decades of the twenty-first century to the Luis Angel Arango Library of the Bank of the Republic of Colombia. The documentary filmmaker, José Miguel Amín Martelo, made an interesting documentary about this donation.^183^

Researchers from Universidad de Antioquia have pioneered studies validating traditional plant-based antidotes used by rural communities, rooted in oral traditions spanning generations.^184–187^ These works bridge indigenous knowledge with phytochemical analysis.

A 2023 meta-analysis coordinated by Universidad Javeriana^188^ cataloged 2199 Colombian herpetology publications (1741–2020), with 70.3% of them post-2000. Most appeared in Spanish-language regional journals, notably *Revista de la Academia Colombiana de Ciencias Exactas, Caldasia, Catálogo de Anfibios y Reptiles de Colombia* and *Zootaxa*. While taxonomy, natural history and biogeography dominate (particularly for Anura and Squamata), the authors stress the need for ecological and conservation-focused studies. In toxinology, J. A. Pereañez and L. A. Preciado^189^ identified 119 Colombian snakebite articles, underscoring the field’s growth.

**Figure 7:**
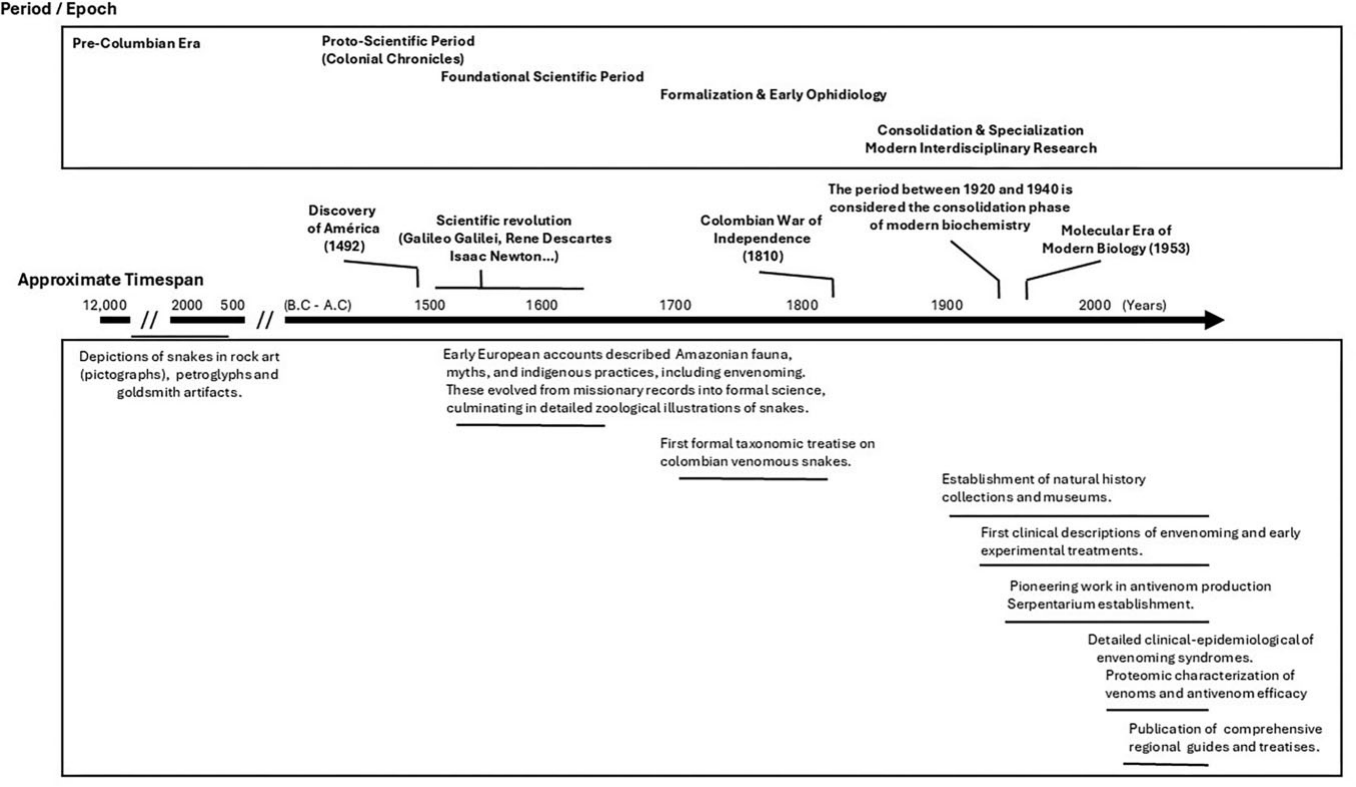
Timeline relating the most relevant aspects related to the sources that support the history of ophidiology and ophidism in Colombia.

Fig. 7 shows the timeline relating the most relevant aspects of the sources that support the history of ophidiology and ophidism in Colombia.

## Perspectives

The long-term viability of snake populations and the preservation of their biodiversity in Colombia and the broader Neotropical region face significant anthropogenic pressures. The primary drivers of this threat are the accelerated loss and fragmentation of critical habitats coupled with the escalating effects of climate change, which collectively alter ecological niches and species distributions.

A consequential public health challenge is the notable increase in human–snake encounters, particularly during seasonal rainfall, as snakes are displaced into peri-urban and urban areas. This elevates the risk of envenomation, a burden compounded by systemic issues in the production, distribution and accessibility of antivenoms. Ophidic envenomation is classified as a neglected tropical disease, and the development of effective therapies is critically constrained by insufficient public health investment and limited commercial incentives for the pharmaceutical industry.

In response to these complex challenges, academic institutions within Colombia and across Latin America are playing an increasingly pivotal role. Their contributions to venom research (spanning toxin characterization, pathophysiology and therapeutic development) are expanding. For Colombia, sustained international collaboration remains a cornerstone of scientific advancement. Strategic partnerships with leading institutions such as Spain’s Institute of Biomedicine of Valencia, Costa Rica’s Clodomiro Picado Institute and Brazil’s Instituto Butantan constitute vital frameworks for research, capacity building and innovation in the field.

## Conclusions

The historical trajectory of ophidian knowledge in Colombia originates with pre-Columbian civilizations, whose empirical observations of indigenous snake species were encoded in iconographic representations and oral traditions. The earliest written accounts emerged during the Spanish colonial era through the Chronicles of the Indies, which documented herpetofaunal encounters in the New World. These narratives, however, were systematically curated and censored by the Council of the Indies, reflecting the geopolitical and doctrinal imperatives of the time. Despite these biases, these chronicles remain invaluable ethnohistorical artifacts.

The post-colonial period and subsequent independence movements catalyzed a paradigm shift towards evidence-based scientific inquiry, yielding foundational contributions to herpetology and toxinology. The nineteenth and twentieth centuries marked exponential advancements in understanding snake biology, venom composition and the pathophysiological mechanisms of envenomation. Contemporary research has further elucidated venom proteomics, clinical envenomation syndromes and evidence-based therapeutic protocols, including antivenom optimization.

Current scientific endeavors prioritize translational research, aiming to exploit venom-derived biomolecules for diagnostic, therapeutic and biotechnological applications. This multidisciplinary continuum (spanning ancestral knowledge, colonial records and modern molecular biology) underscores Colombia’s pivotal role in advancing global ophidism while addressing regional public health challenges posed by snake encounters.

## Data Availability

The data underlying this article will be shared upon reasonable request to the corresponding author.
